# Incidence, predictability, and outcomes of systemic venous congestion following a fluid challenge in initially fluid-tolerant preload-responders after cardiac surgery: a pilot trial

**DOI:** 10.1186/s13054-024-05124-6

**Published:** 2024-10-22

**Authors:** Bianca Morosanu, Cosmin Balan, Cristian Boros, Federico Dazzi, Adrian Wong, Francesco Corradi, Serban-Ion Bubenek-Turconi

**Affiliations:** 1 1st Department of Cardiovascular Anesthesia and Intensive Care Medicine, Prof. Dr. C.C. Iliescu Institute for Emergency Cardiovascular Diseases, 022328 Bucharest, Romania; 2 Unit of Cardiac Anesthesia and Intensive Care, Fondazione Toscana Gabriele Monasterio, Hospital of Massa, Pisa, Italy; 3 Department of Critical Care, King’s College Hospital, London, UK; 4 Department of Surgical, Medical, Molecular Pathology and Critical Care Medicine, University of Pisa, Pisa, Italy

**Keywords:** Fluid resuscitation, Preload responsiveness, Fluid tolerance, Systemic venous congestion, Portal vein pulsatility index

## Abstract

**Background:**

Fluid administration has traditionally focused on preload responsiveness (PR). However, preventing fluid intolerance, particularly due to systemic venous congestion (VC), is equally important. This study evaluated the incidence and predictability of VC following a 7 ml/kg crystalloid infusion in fluid-tolerant preload-responders and its association with adverse outcomes.

**Methods:**

This single-center, prospective, observational study (May 2023–July 2024) included 40 consecutive patients who were mechanically ventilated within 6 h of intensive care unit (ICU) admission after elective open-heart surgery and had acute circulatory failure. Patients were eligible if they were both fluid-tolerant and preload-responsive. PR was defined as a ≥ 12% increase in left-ventricular outflow tract velocity time integral (LVOT-VTI) 1 min after a passive leg raising (PLR) test. VC was defined by a portal vein pulsatility index (PVPI) ≥ 50%. Patients received a 7 ml/kg Ringer’s Lactate infusion over 10 min. The primary outcome was the incidence of VC 2 min post-infusion (early-VC). Secondary outcomes included VC at 20 min, the incidence of acute kidney injury (AKI) and severe AKI at 7 days, and ICU length of stay (LOS).

**Results:**

45% of patients developed early-VC, with VC persisting in only 5% at 20 min. One-third of patients developed AKI, with 17.5% progressing to severe AKI. The median ICU LOS was 4 days. Patients with early-VC had significantly higher central venous pressure, lower mean perfusion pressure, worse baseline right ventricular function, and a higher incidence of severe AKI. While LVOT-VTI returned to baseline by 20 min in both groups, PVPI remained elevated in early-VC patients (*p* < 0.001). The LVOT-VTI versus PVPI regression line showed similar slopes (*p* = 0.755) but different intercepts (*p* < 0.001), indicating that, despite fluid tolerance and PR at baseline, early-VC patients had reduced right ventricular diastolic reserve (RVDR). Post-PLR PVPI predicted early-VC with an area under the curve of 0.998, using a threshold of 44.3% (*p* < 0.001).

**Conclusion:**

Post-PLR PVPI effectively predicts fluid-induced early-VC in fluid-tolerant preload-responders, identifying those with poor RVDR. Its use can guide fluid management in cardiac surgery patients, helping to prevent unnecessary fluid administration and associated complications.

*Trial Registration*: NCT06440772. Registered 30 May 2024. Retrospectively registered.

## Background

Administering fluids is crucial for maintaining adequate tissue perfusion, balancing the prevention of organ ischemia and congestion [1]. Traditionally, fluids are given to preload-responders with cardiocirculatory failure to increase stroke volume. However, maximizing stroke volume until fluid unresponsiveness is achieved should not be the clinician’s goal, as this approach can exceed safe ventricular filling pressures, risk interstitial leakage, and create an unphysiological hemodynamic state by depleting the preload reserve [2]. Additionally, it may cause hemodilution, potentially hindering the expected increase in oxygen delivery relative to the increase in cardiac output [3].

Fluid tolerance—how well a patient can handle fluids without causing organ congestion—may be compromised when hydrostatic pressures are increased to increase stroke volume [4]. Increased capillary permeability and low oncotic pressure can further exacerbate tissue edema. Organ congestion can be central (lung), systemic, or mixed [5]. Even patients identified as preload-responders may experience fluid intolerance, as indicated by systemic congestion markers such as central venous pressure (CVP) and the Venous EXcess UltraSound (VEXUS) score, or central/lung congestion markers such as the ratio of early diastolic mitral inflow velocity to mitral annular early diastolic velocity (E/e′) and the lung ultrasound score [6]. Thus, preload responsiveness does not guarantee fluid tolerance, as these two distinct functional determinants can overlap, with fluid intolerance often occurring before the preload reserve is fully depleted.

To reduce adverse events, the paradigm should shift to administering fluids only to those who are both preload-responders and fluid-tolerant at baseline [5]. However, the incidence, predictability, and clinical significance of developing systemic venous congestion after a routine crystalloid challenge remain unexplored.

Studies have shown that a portal vein pulsatility index (PVPI) exceeding 50% is associated with increased postoperative complications and prolonged life support in high-risk cardiac surgery patients [7–9]. In the absence of a gold standard for diagnosing congestive organ injury, this association is thought to reflect systemic venous congestion [8]. For the purposes of this study, fluid intolerance is attributed to clinically significant systemic venous congestion, identified by a PVPI ≥ 50%.

This study examined the incidence and predictability of systemic venous congestion following a 7 ml/kg crystalloid challenge in initially fluid-tolerant preload-responders, and its association with adverse clinical outcomes.

## Material and methods

### Study design and ethics

This single-center, prospective, observational clinical study was conducted from May 2023 to July 2024 at the 1st Department of Cardiovascular Anesthesia and Intensive Care Medicine, Prof. Dr. C. C. Iliescu Institute for Emergency Cardiovascular Diseases, Bucharest, Romania. Informed consent was obtained from all participants, and their identities were kept confidential during data analysis. The study adhered to the Declaration of Helsinki, received approval from the local Institutional Review Board for Biomedical Research (reference number 6066/21 February 2023), and was retrospectively registered at ClinicalTrials.gov as NCT06440772 on 30 May 2024. The study's presentation follows the STROBE checklist.

### Patient selection

All patients were mechanically ventilated within 6 h of ICU admission following elective open-heart surgery. We specifically recruited patients with acute circulatory failure who were both preload-responsive and fluid-tolerant before administering 7 ml/kg of Ringer’s Lactate over 10 min. Acute circulatory failure was defined by clinical signs of hypoperfusion (e.g., mottled skin, oliguria), elevated lactate levels (> 2 mmol/l), reduced central venous oxygen saturation (ScVO_2_ < 70%), and an increased difference between central venous and arterial partial pressures of carbon dioxide (ΔCO_2_ > 6 mmHg), with or without concomitant hypotension (systolic blood pressure < 90 mm Hg or mean arterial pressure (MAP) < 65 mm Hg) [10]. Patients were in sinus rhythm, equipped with central venous and arterial catheters, sedated, and on controlled mechanical ventilation. Settings included a positive end-expiratory pressure (PEEP) of 5 cmH2O, a tidal volume of 6–8 ml/kg ideal body weight, and a fraction of inspired oxygen (FiO_2_) adjusted to maintain arterial oxygen saturation (SaO_2_) at 96–98%, with a respiratory rate set to maintain CO_2_ levels at 35–40 mmHg.

The exclusion criteria included: (a) age under 18 years, (b) preload unresponsiveness or fluid intolerance, (c) conditions interfering with portal vein flow assessment or interpretation (e.g., liver cirrhosis, chronic hepatic disease, suprahepatic/portal vein thrombosis), (d) mechanical circulatory support, (e) cardiac transplant, (f) poor echocardiographic window, and (g) previous amputation. Eligible patients underwent simultaneous echocardiographic and clinical assessments.

### Echocardiographic measurements

Transthoracic echocardiography was performed via a Philips CX50 system equipped with a 2.0–4.0 MHz S4–2 Broadband Sector Array Transducer (Koninklijke Philips N.V., Eindhoven, Netherlands), and electrocardiogram gating. Prior to the examination, sedation was adjusted to a Richmond Agitation Sedation Scale score of − 3/− 4 and a neuromuscular blocker bolus was administered to ensure passive ventilation. Measurements, averaged from three consecutive end-expiratory readings, were conducted by a single trained operator (B.M.) and later reviewed by a certified echocardiography physician (C.BA.) for validation.

#### Assessment of the preload responsiveness state

Passive leg raising (PLR) was employed to assess preload responsiveness, with the patient positioned semi-recumbently in accordance with established protocols [11]. The left ventricular outflow tract velocity–time integral (LVOT-VTI) was measured using pulsed wave (PW) Doppler from a five-chamber apical view. Patients were classified as preload responders if the LVOT-VTI increased by at least 12% 1 min after PLR and returned to baseline when the semi-recumbent position was resumed. The 12% cutoff was selected on the basis of the least significant change (LSC) of 11% (range 5–18%) between consecutive measurements by the same operator, as reported by Jozwiak et al. [12].

#### Assessment of the fluid tolerance state

The portal vein pulsatility index (PVPI) was measured using pulsed wave (PW) Doppler on the portal vein at the liver hilum. The PVPI was calculated by recording the maximum (Vmax) and minimum (Vmin) velocities and applying the formula: PVPI = (Vmax − Vmin)/Vmax [13]. A PVPI < 50 indicated fluid tolerance, whereas a PVPI ≥ 50 signified fluid intolerance and systemic venous congestion. Intra-observer reproducibility for PVPI was assessed by calculating precision as 2 × CE (coefficient of error), where CE is defined as CV/√n [12, 14]. Here, CV refers to the coefficient of variation (discussed later), and n is the minimum theoretical number of replicates. The median precision was 8.2% (7.2–11.8). Notably, only intra-observer reproducibility was evaluated, as all measurements were performed by a single operator (B.M.).

#### Additional assessments

Other baseline transthoracic echocardiography variables were included as per recent recommendations [15]: (1) left ventricular ejection fraction (LVEF) measured by Simpson’s biplane method; (2) the ratio of right to left ventricular end-diastolic areas (RVEDA/LVEDA); (3) tricuspid annular plane systolic excursion (TAPSE); (4) right ventricular fractional area change (RVFAC); (5) tricuspid lateral annular systolic velocity (RV S’) derived from tissue Doppler; (6) maximum diameter of the inferior vena cava (IVC); and (7) IVC distensibility index, calculated as (Dmaximum at end-inspiration − Dminimum at end-expiration)/Dminimum at end-inspiration.

### Clinical data collection

Clinical data included demographic information; macrohemodynamic variables (MAP, CVP, and mean perfusion pressure (MPP), calculated as the difference between MAP and CVP); vasoactive drug use; and severity scores, such as the admission Sequential Organ Failure Assessment (SOFA) score and the preoperative European System for Cardiac Operative Risk Evaluation II (EuroSCORE II). Baseline creatinine levels, tissue perfusion-related variables (arterial lactate, ScVO_2_, and ΔCO_2_), ICU length of stay (ICU LOS), and the development of acute kidney injury (AKI) by day 7 were also recorded.

AKI was defined according to the Kidney Disease Improving Global Outcomes (KDIGO) criteria as an increase in serum creatinine of ≥ 0.3 mg/dl within a 48-h period or a 50% increase from baseline creatinine within a week from cardiac surgery. Severe AKI was defined as an increase of 100% or more in baseline creatinine, a serum creatinine level of ≥ 4 mg/dl, or the initiation of renal replacement therapy (KDIGO stage 2 or 3) [16].

### Study protocol

Within 6 h of ICU admission following elective cardiac surgery with cardiopulmonary bypass, patients with acute circulatory failure were screened for eligibility. The study proceeded as follows:

*T1* (Baseline): Baseline echocardiographic and clinical data, including assessments of fluid tolerance, were collected.

*T2* (Post-PLR): LVOT-VTI and PVPI were measured 1 min after PLR, and clinical data were collected.

*T3* (Return from PLR): Patients were returned to their original position, and after 2 min, the LVOT-VTI, PVPI, and clinical data were collected.

If patients were both fluid-tolerant at baseline and preload-responsive after PLR, they received 7 ml/kg Ringer’s Lactate over 10 min.

*T4* (Post-Ringer’s Lactate): PVPI and LVOT-VTI were measured 2 min after the completion of the crystalloid challenge, and clinical data were collected.

*T5* (20 min post-Ringer’s Lactate): PVPI and LVOT-VTI were measured again 20 min after the completion of the crystalloid challenge, and clinical data were collected.

The 2-min intervals at T3 and T4 for data collection were chosen on the basis of findings from previously published studies [17]. No additional fluids were administered, and vasoactive drug regimens remained unchanged throughout the T1 to T5 transitions.

### Outcomes

The primary outcome was the incidence of systemic venous congestion defined as a PVPI ≥ 50, which was assessed 2 min after the administration of 7 ml/kg Ringer’s Lactate over 10 min. The secondary outcomes included systemic venous congestion at 20 min postinfusion, the incidence of AKI and severe AKI at 7 days, and the ICU LOS. An exploratory analysis was also performed to predict systemic venous congestion.

### Statistics

Statistical analyses were performed using NCSS 2024 Statistical Software (NCSS, LLC, Kaysville, Utah, USA), Stata/BE 18.0 (StataCorp, College Station, Texas, USA) and R Statistical Software (v. 4.3.0; R Core Team, 2023) depending on the specific requirements of the analysis. The normality of quantitative variables was assessed through visual inspection and the Shapiro–Wilk test. Normally distributed data are presented as means ± standard deviations (SDs), while non-normally distributed data are reported as medians with interquartile ranges (IQRs, 25th-75th percentiles). Categorical data are summarized as frequencies and percentages.

Nonparametric quantitative outcomes were evaluated using the Mann–Whitney U test, while binary outcomes were compared using either the chi-squared test or Fisher’s exact test, depending on sample size. Receiver operating characteristic (ROC) curve analysis was performed using bootstrapping with 1000 iterations, each generating a ROC curve with 40 data points. This method facilitated the construction of mean ROC curves and the calculation of key metrics such as sensitivity, specificity, positive predictive value, negative predictive value, and the area under the curve (AUC), all with their respective 95% confidence intervals. Comparisons between ROC curves were made by analyzing differences in AUCs across bootstrapped samples, deriving mean differences, confidence intervals, and *p*-values directly from the bootstrap distributions. Grey zone identification used established methodologies to identify values where sensitivity or specificity fall below 90% and to calculate confidence intervals for cutoffs derived from the optimal Youden index [18].

A mixed model for repeated measures (MMRM) analysis was conducted to assess overall and group-specific trends of LVOT-VTI and PVPI across five timepoints (T1 to T5). This model used a diagonal covariance structure with random intercepts for patients to minimize the Akaike information criterion (AIC). Fixed effects included group (presence or absence of systemic venous congestion following the fluid challenge), time, and their interaction, with results presented as least squares adjusted (LSA) means ± standard errors (SEs) and 95% confidence intervals (CIs). Next, the correlation (ρ) between repeated measurements of LVOT-VTI and PVPI was analyzed using the repeated measure correlation (rmcorr) package. Finally, an MMRM analysis was used to compare the slopes and intercepts of regression lines for group-specific trends between PVPI and LVOT-VTI, evaluating differences in their relationships over time.

In the MMRM analyses, Bonferroni correction was applied to adjust the observed *p*-value for multiple comparisons, with significance established at an adjusted *p*-value of less than 0.05. Fisher’s z-test was applied to compare the correlation coefficients between the groups. For all other statistical tests, a significance level of *p* < 0.05 (two-tailed) was used.

No power analysis was conducted due to the exploratory nature of this pilot study and the absence of prior relevant data.

## Results

**Fig. 1:**
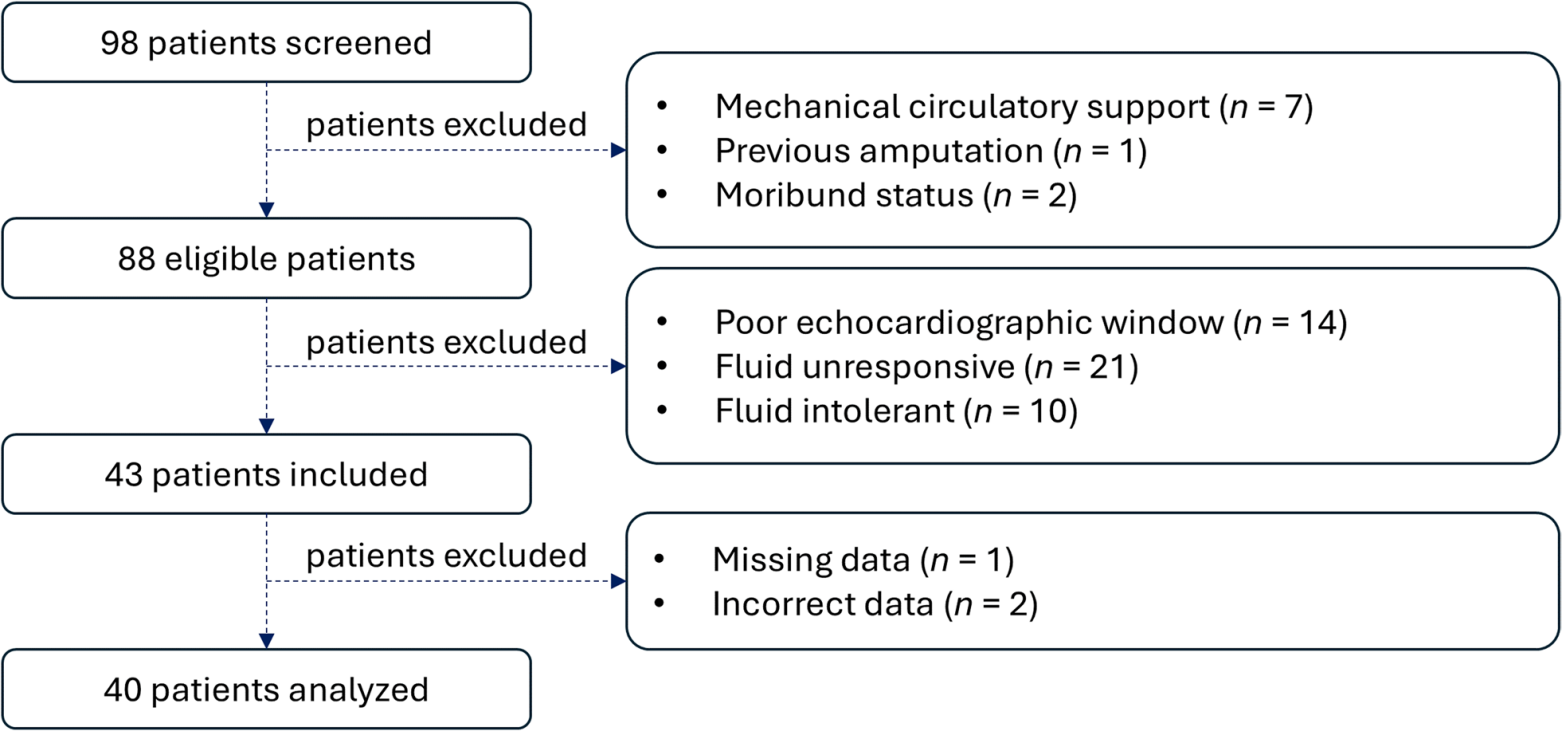
Study flow chart

Among the 98 patients screened, 55 met the exclusion criteria, and 3 were excluded because of missing or incorrect data, resulting in 40 patients for the final analysis (Fig. 1).

Patients had a median age of 62 years, with nearly two-thirds being male and having undergone valvular surgery. The median EuroSCORE II was 2.6, the mean SAPS II was 4.2, and the mean MPP was 70.6 mmHg. Patients were on low doses of norepinephrine and dobutamine, with a median LVEF of 50%, a median TAPSE of 15 mm, and a mean RVFAC of 35.1%. Following the infusion of Ringer’s Lactate, 45% of the patients (18 out of 40) developed congestion (PVPI ≥ 50), which persisted at 20 min in only 5% (2 out of those 18) of the patients. One-third of the patients developed AKI at any stage, with 17.5% progressing to severe AKI (KDIGO stage 2 or 3) at 7 days. The median ICU LOS was 4 days (Table 1).

**Table 1:** Baseline demographic variables, perioperative data, and outcomes of the patients included

Parameter	All patients (*n* = 40)
Demographics	
Age, years	62.0 (55.5–68.0)
Gender, male	27 (67.5)
BMI, kg/m^2^	28.7 ± 4.6
Weight, kg	82.5 ± 13.4
Surgery characteristics	
Total duration, min	168.0 (150.3–196.5)
CPB time, min	88.0 (70.3–116.5)
ACC time, min	63.5 (50.5–81.3)
Type of surgery	
Valvular surgery	25 (62.5)
CABG (± valves)	12 (30.0)
Miscellaneous	3 (7.5)
Perioperative scores and kidney function	
EuroSCORE II, % mortality	2.6 (1.3–4.3)
SAPS II (6-h ICU), % mortality	4.2 ± 1.7
Preoperative creatinine, mg/dl	0.9 (0.8–1.1)
Preoperative CrCl, ml/min	95.7 ± 29.4
Perfusion variables at T1	
MAP, mmHg	77.5 ± 7.8
CVP, mmHg	6.9 ± 2.5
MPP, mmHg	70.6 ± 8.6
Lactate, mmol/L	2.4 ± 0.8
ScVO_2_, %	72.3 ± 8.5
ΔCO_2_, mmHg	9.8 ± 4.5
Vasoactive drugs at T1	
Norepinephrine, ng kg^−1^ min^−1^	37.0 (26.0–54.3)
Dobutamine, μg kg^−1^ min^−1^	5.0 (3.0–5.6)
Echocardiographic variables at T1	
LVEF, %	50.0 (45.0–58.0)
RVFAC, %	35.1 ± 7.6
RVEDA/LVEDA ratio	0.5 ± 0.1
TAPSE, mm	15.0 (13.0–17.0)
RV S’, cm/s	9.0 (7.6–10.3)
IVC Dmaximum, cm	18.0 (16.3–19.8)
IVC DI, %	12.1 (5.9–20.0)
Outcomes	
Congestion (PVPI ≥ 50%) at T4	18 (45.0)
Congestion (PVPI ≥ 50%) at T5	2 (5.0)
AKI (KDIGO 1–3) at 7 days	13 (32.5)
Severe AKI (KDIGO 2–3) at 7 days	7 (17.5)
ICU LOS, days	4 (3–4)

Values are presented as mean ± SD, median (IQR, 25th-75th percentiles) or counts (percentages)

*ACC* aortic cross-clamp, *AKI* acute kidney injury, *BMI* body mass index, *CABG* coronary artery bypass graft, *CPB* cardiopulmonary bypass, *CrCl* creatinine clearance, *CVP* central venous pressure, *ΔCO*_2_ difference between central venous partial pressure of carbon dioxide (PCO_2_) and arterial PCO_2_, *EuroSCORE II* European System for Cardiac Operative Risk Evaluation, *ICU LOS* intensive care unit length of stay, *IVC Dmaximum* maximum diameter of inferior vena cava, *IVC DI* IVC distensibility index, *KDIGO* Kidney Disease: Improving Global Outcomes, *LVEF* left ventricular ejection fraction, *LVEDA* left ventricular end-diastolic area, *MAP* mean arterial pressure, *MPP* mean perfusion pressure, *PVPI* portal vein pulsatility index, *RVEDA* right ventricular end-diastolic area, *RVFAC* right ventricular fractional area change, *RV S’* tissue Doppler-derived tricuspid lateral annular systolic velocity, *SAPS II* simplified acute physiology score, *ScVO*_2_ central venous oxygen saturation, *T1* baseline, *T4* 2 min post-Ringer’s Lactate, *T5* 20 min post-Ringer’s Lactate, *TAPSE* tricuspid annular plane systolic excursion

**Fig. 2:**
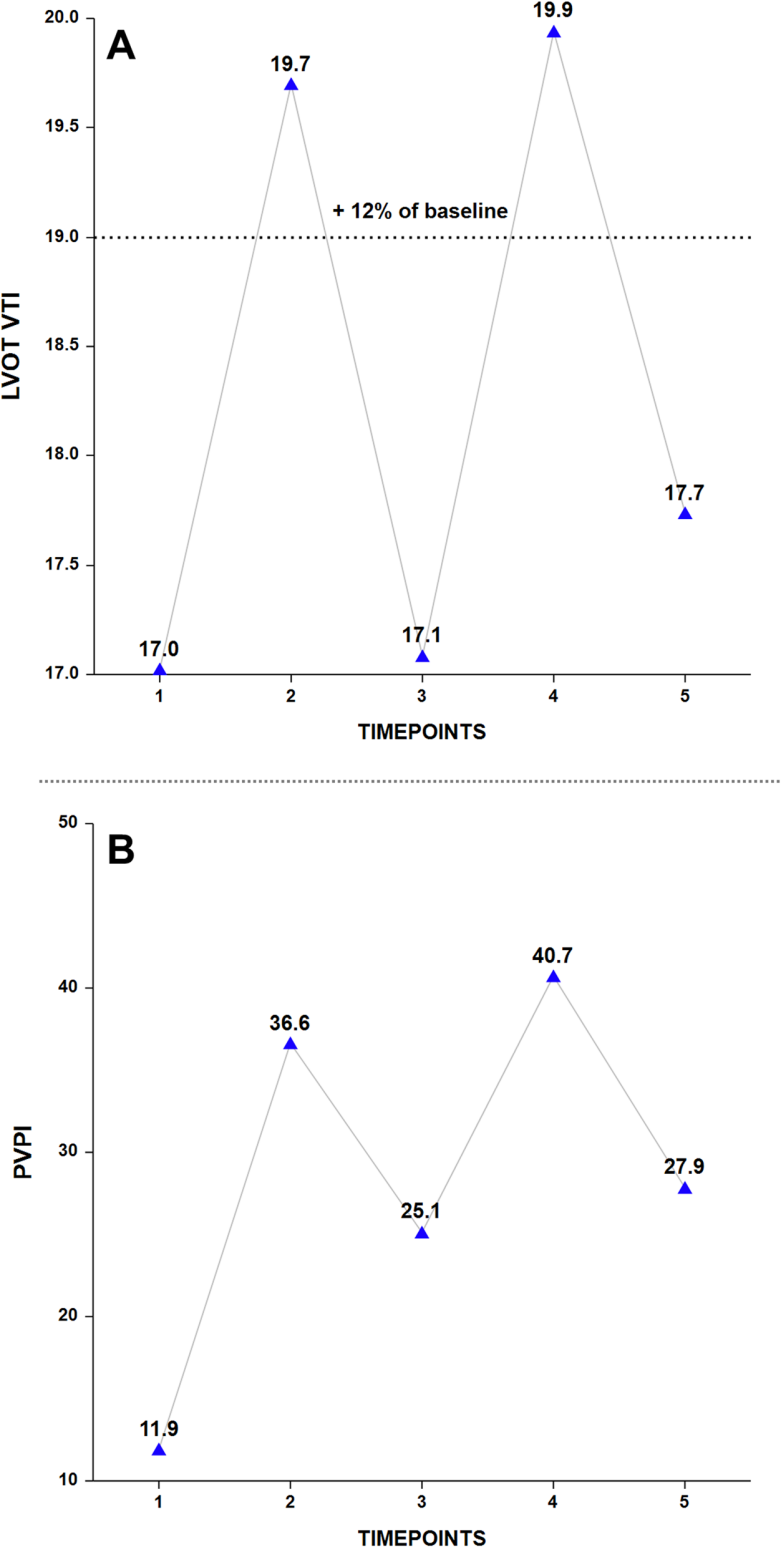
LVOT-VTI and PVPI trends in all patients. Values are presented as LSA means. *LSA* least squares adjusted, *LVOT-VTI* left ventricular outflow tract velocity time integral, *PVPI* portal vein pulsatility index, *T1* baseline, *T2* 1 min after passive leg raising, *T3* 2 min after return to the semi-recumbent position, *T4* 2 min post-Ringer’s Lactate, *T5* 20 min post-Ringer’s Lactate

A longitudinal analysis using an MMRM design revealed significant main effects for time in both flow (LVOT-VTI) (F-value = 117.554, *p* < 0.001) and congestion (PVPI) (F-value = 88.483, *p* < 0.001) (Fig. 2, Table 2). The baseline value (T1) was the nadir for both metrics. Although the T5 LVOT-VTI was still significantly greater than T1, the relative increase dropped from 17% at T4 to just 4% at T5, falling below the 12% threshold for fluid responsiveness. This reduction underscores the short-term effect of a 7 ml/kg crystalloid challenge. In contrast, the congestion signal (PVPI) peaked at 3.4 times the baseline at T4 and remained elevated at 2.3 times the baseline at T5.

**Table 2:** LVOT-VTI and PVPI metrics for all patients

Timepoint	LVOT-VTI		PVPI	
	Value	*p*-value	Value	*p*-value
T1	17.0 ± 0.4 (16.2–17.8)	NA	11.9 ± 2.6 (6.8–17.0)	NA
T2	19.7 ± 0.4 (18.9–20.5)	T2-to-T1: < 0.001	36.6 ± 2.6 (31.5–41.7)	T2-to-T1: < 0.001
T3	17.1 ± 0.4 (16.3–17.9)	T3-to-T1: 1.000	25.1 ± 2.6 (20.0–30.3)	T3-to-T1: < 0.001
T4	19.9 ± 0.4 (19.1–20.8)	T4-to-T1: < 0.001	40.7 ± 2.6 (35.6–45.8)	T4-to-T1: < 0.001
T5	17.7 ± 0.4 (16.9–18.6)	T5-to-T1: 0.002	27.9 ± 2.6 (22.7–33.0)	T5-to-T1: < 0.001

Values are presented as LSA means ± SE and 95% confidence intervals (CI) for LSA means

*LSA* least squares adjusted, *LVOT-VTI* left ventricular outflow tract velocity time integral, *PVPI* portal vein pulsatility index, *T1* baseline, *T2* 1 min after passive leg raising, *T3* 2 min after return to semi-recumbent position, *T4* 2 min post-Ringer’s Lactate, *T5* 20 min post-Ringer’s Lactate

A second longitudinal analysis using an MMRM design compared flow (LVOT-VTI) and congestion (PVPI) trends in the initial cohort, which were divided into two groups by congestion status 2 min post-Ringer’s Lactate infusion (T4): 18 patients with PVPI ≥ 50% (fluid-intolerant, indicating systemic venous congestion) and 22 patients with PVPI < 50% (fluid-tolerant, without systemic venous congestion).

**Fig. 3:**
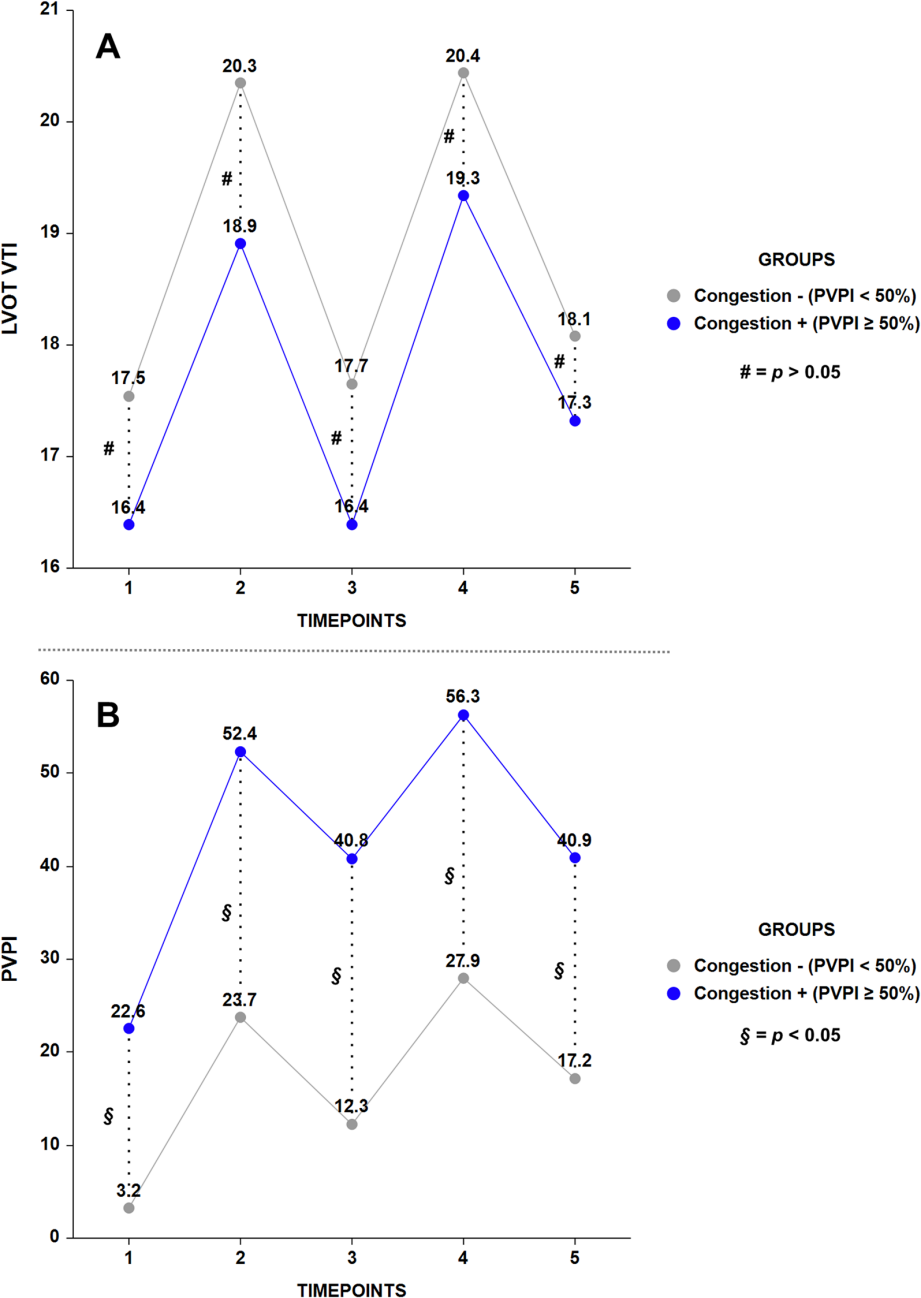
Group-specific comparison of LVOT-VTI and PVPI trends. Values are presented as LSA means. *LSA* least squares adjusted, *LVOT-VTI* left ventricular outflow tract velocity time integral, *PVPI* portal vein pulsatility index, *T1* baseline, *T2* 1 min after passive leg raising, *T3* 2 min after return to semi-recumbent position, *T4* 2 min post-Ringer’s Lactate, *T5* 20 min post-Ringer’s Lactate

For LVOT-VTI, significant effects were observed for time (F = 115.580, *p* < 0.001), but not for group (F = 2.174, *p* = 0.149) or the time-group interaction (F = 0.922, *p* = 0.453) (Fig. 3A, Table 3). In contrast, for the PVPI, significant effects were observed for time (F = 95.242, *p* < 0.001), group (F = 131.175, *p* < 0.001), and the time-group interaction (F = 3.132, *p* < 0.001) (Fig. 3B, Table 3). Fluid-intolerant patients at T4 had significantly greater PVPI values at all timepoints, whereas the LVOT-VTI values remained statistically similar between the groups throughout the study period. The 7 ml/kg fluid challenge briefly improved arterial flow in both groups, with the effect dissipating equally by 20 min, but it resulted in an impaired venous flow as indicated by a persistently and significantly greater PVPI in one group.

**Table 3:** Group-specific comparison of LVOT-VTI and PVPI metrics

Timepoint	LVOT-VTI			PVPI		
	No congestion (n = 22)	Congestion (n = 18)	*p*-value	No congestion (n = 22)	Congestion (n = 18)	*p*-value
T1	17.5 ± 0.5 (16.4–18.6)	16.4 ± 0.6 (15.2–17.6)	0.803	3.2 ± 2.1 (0.9–7.3)	22.6 ± 2.3 (18.1–27.1)	< 0.001
T2	20.3 ± 0.5 (19.3–21.4)	18.9 ± 0.6 (17.7–20.1)	0.410	23.7 ± 2.1 (19.7–27.8)	52.4 ± 2.3 (47.9–56.9)	< 0.001
T3	17.7 ± 0.5 (16.6–18.7)	16.4 ± 0.6 (15.2–17.6)	0.629	12.3 ± 2.1 (8.2–16.3)	40.8 ± 2.3 (36.3–45.3)	< 0.001
T4	20.4 ± 0.5 (19.3–21.5)	19.3 ± 0.6 (18.1–20.5)	0.909	27.9 ± 2.1 (23.8–32.0)	56.3 ± 2.3 (51.8–60.8)	< 0.001
T5	18.1 ± 0.5 (17.0–19.2)	17.3 ± 0.6 (16.1–18.5)	1.000	17.2 ± 2.1 (13.1–21.3)	40.9 ± 2.3 (36.4–45.4)	< 0.001

Values are presented as LSA means ± SE and 95% confidence intervals (CI) for LSA means

*LSA* least squares adjusted, *LVOT-VTI* left ventricular outflow tract velocity time integral, *PVPI* portal vein pulsatility index, *T1* baseline, *T2* 1 min after passive leg raising, *T3* 2 min after return to semi-recumbent position, *T4* 2 min post-Ringer’s Lactate, *T5* 20 min post-Ringer’s Lactate

**Fig. 4:**
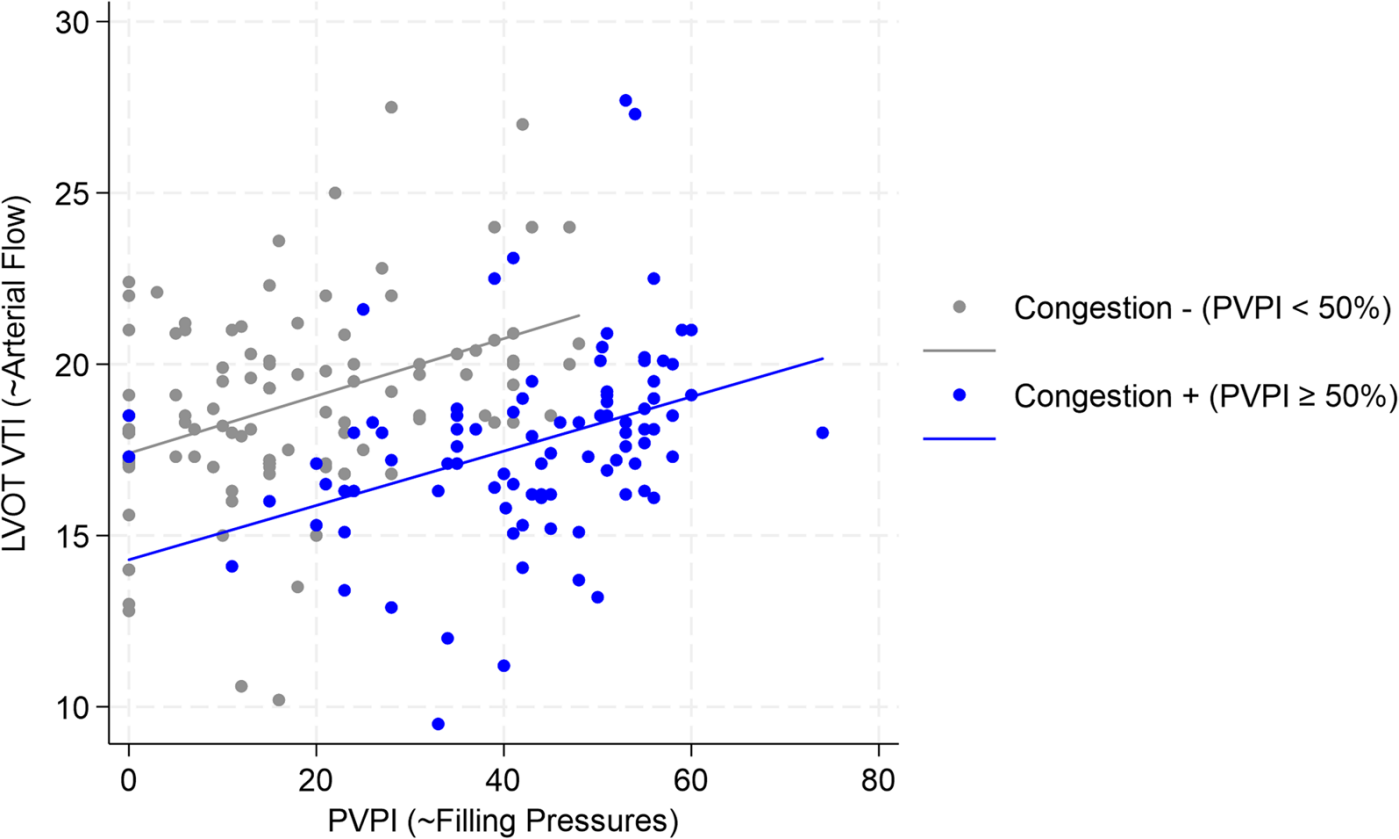
Group-Specific LVOT-VTI Regression Lines as a Function of PVPI. For the no congestion versus congestion groups, the repeated measures correlation coefficients (ρ) were 0.622 (95% CI 0.473–0.736) versus 0.738 (95% CI 0.609–0.828) (*p* = 0.568); the intercepts for LVOT-VTI at PVPI = 0 were 17.40 (95% CI 16.37–18.43) versus 14.29 (95% CI 12.94–15.62) (*p* < 0.001); the slopes, representing the change in LVOT-VTI per unit increase in PVPI, were 0.084 (95% CI 0.062–0.105) versus 0.079 (95% CI 0.063–0.096) (*p* = 0.755). *LVOT-VTI* left ventricular outflow tract velocity time integral, *PVPI* portal vein pulsatility index

The relationship between arterial flow and congestion was assessed using rmcorr analysis, revealing a strong correlation between LVOT-VTI and PVPI in both groups: ρ = 0.622 (*p* < 0.001) in non-congestive patients and ρ = 0.738 (*p* < 0.001) in congestive patients. These correlation coefficients are visualized in the mixed-effects model graph (Fig. 4), which also includes group-specific regression lines, illustrating how the relationship between PVPI and LVOT-VTI varies over time across subjects and groups. Notably, the two groups differed significantly in intercepts (*p* < 0.001) but not in slopes (*p* = 0.755), indicating that at the same PVPI, the congestive group is likely to have a lower LVOT-VTI, and thus, reduced arterial flow.

The two groups were compared in terms of key perioperative, hemodynamic, echocardiographic, and outcome-related variables (Table 4). No significant differences were found between the groups regarding age, surgical characteristics, preoperative risk, renal function, MAP, lactate, ScVO_2_, ΔCO_2_, left ventricular performance, vasoactive drug use, ICU LOS, or AKI incidence at 7 days. However, patients with PVPI ≥ 50% at 2 min post-Ringer’s Lactate infusion had higher CVP and lower MPP, along with poorer right ventricular function (indicated by lower RVFAC, TAPSE, and RV S’, a higher RVEDA/LVEDA ratio, and a larger IVC with reduced distensibility), a higher SAPS II score, and a greater incidence of severe AKI at 7 days.

**Table 4:** Comparison of key perioperative, hemodynamic, echocardiographic, and outcome-related variables

Parameter	No congestion (n = 22)	Congestion (n = 18)	*p*-value
Demographics			
Age, years	63.5 (58.8–69.0)	60.5 (51.5–68.5)	0.446
Surgery characteristics			
Total duration, min	172.0 (157.3–198.3)	158.0 (148.0–209.0)	0.541
CPB time, min	92.0 (77.3–118.3)	78.0 (68.0–129.0)	0.550
ACC time, min	62.0 (52.8–79.8)	64.0 (48.5–97.5)	0.946
Perioperative scores and kidney function			
EuroSCORE II, % mortality	2.0 (1.1–3.6)	3.2 (2.0–4.7)	0.100
SAPS II (6-h ICU), % mortality	3.4 ± 1.2	5.2 ± 1.8	0.001
Preoperative creatinine, mg/dl	0.9 (0.8–1.1)	0.8 (0.7–1.1)	0.541
Preoperative CrCl, ml/min	91.2 ± 28.4	101.3 ± 30.5	0.314
Perfusion variables at T1			
MAP, mmHg	79.3 ± 7.7	75.3 ± 7.6	0.109
CVP, mmHg	6.2 ± 2.2	7.8 ± 2.6	0.035
MPP, mmHg	73.1 ± 7.9	67.4 ± 8.6	0.037
Lactate, mmol/l	2.3 ± 0.8	2.5 ± 0.8	0.449
ScVO_2_, %	73.6 ± 8.8	70.8 ± 8.0	0.307
ΔCO_2_, mmHg	8.6 ± 4.0	11.2 ± 4.7	0.077
Vasoactive drugs at T1			
Norepinephrine, ng kg^−1^ min^−1^	30.0 (20.0–50.0)	50.0 (30.0–70.0)	0.077
Dobutamine, μg kg^−1^ min^−1^	5.0 (3.0–7.3)	5.0 (3.0–5.0)	0.390
Echocardiographic variables at T1			
LVEF, %	50.5 (49.8–59.4)	48.8 (44.5–55.0)	0.138
RVFAC, %	37.7 ± 6.7	31.8 ± 7.5	0.014
RVEDA/LVEDA ratio	0.5 ± 0.1	0.6 ± 0.1	< 0.001
TAPSE, mm	15.6 (14.0–17.5)	13.0 (13.0–15.3)	0.045
RV S’, cm/s	10.0 (8.6–10.5)	8.4 (7.3–9.2)	0.003
IVC Dmaximum, cm	17.0 (15.0–18.0)	20.0 (19.0–21.0)	< 0.001
IVC DI, %	19.2 (12.3–27.1)	5.9 (1.6–11.8)	< 0.001
Outcomes			
AKI (KDIGO 1–3) at 7 days	5 (22.7)	8 (44.4)	0.145
Severe AKI (KDIGO 2–3) at 7 days	1 (4.5)	6 (33.3)	0.033
ICU LOS, days	3 (3.0–4.0)	4 (3.0–5.0)	0.111

Values are presented as mean ± SD, median (IQR, 25th-75th percentiles) or counts (percentages)

*ACC* aortic cross-clamp, *AKI* acute kidney injury, *CPB* cardiopulmonary bypass, *CrCl* creatinine clearance, *CVP* central venous pressure, *ΔCO*_2_ difference between central venous partial pressure of carbon dioxide (PCO_2_) and arterial PCO2, *EuroSCORE II* European System for Cardiac Operative Risk Evaluation, *ICU LOS* intensive care unit length of stay, *IVC Dmaximum* maximum diameter of inferior vena cava, *IVC DI* IVC distensibility index, *KDIGO* Kidney Disease: Improving Global Outcomes, *LVEF* left ventricular ejection fraction, *LVEDA* left ventricular end-diastolic area, *MAP* mean arterial pressure, *MPP* mean perfusion pressure, *PVPI* portal vein pulsatility index, *RVEDA* right ventricular end-diastolic area, *RVFAC* right ventricular fractional area change, *RV S’* tissue Doppler-derived tricuspid lateral annular systolic velocity, *SAPS II* Simplified acute physiology score, *ScVO*_2_ central venous oxygen saturation, *T1* baseline, *TAPSE* tricuspid annular plane systolic excursion

**Fig. 5:**
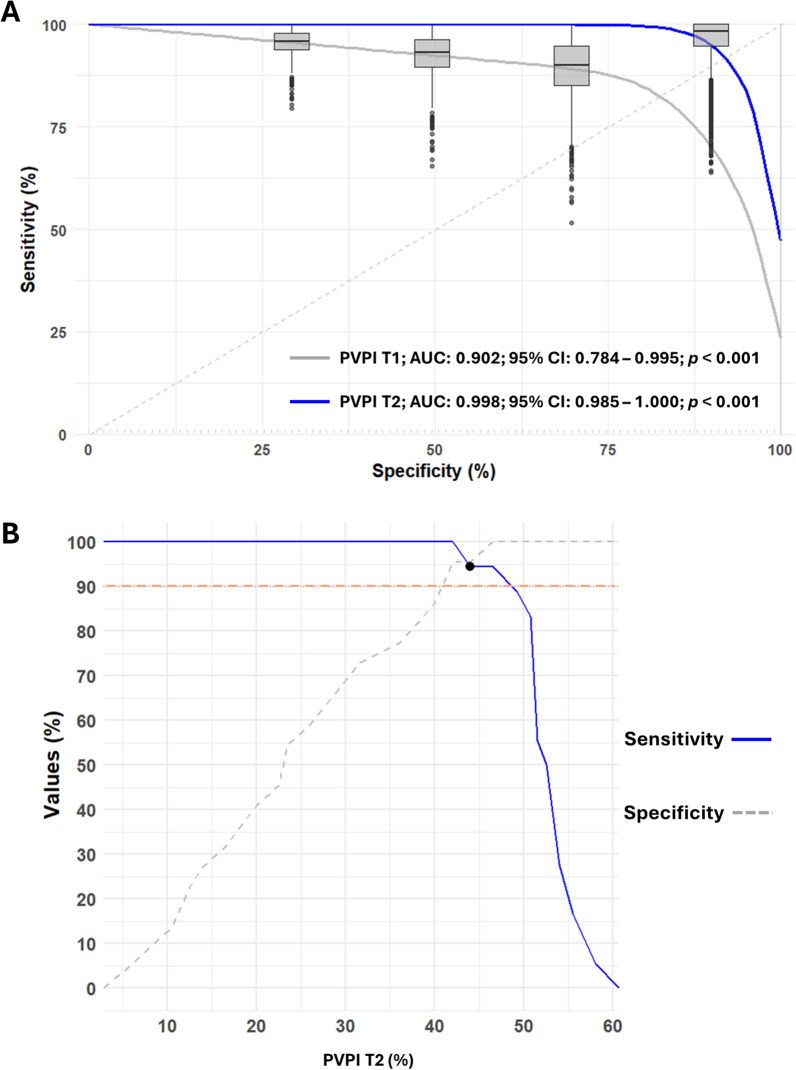
Bootstrapped ROC curve analysis for predicting T4 congestion. **A** Discriminative power of PVPI at T1 and T2 with box plots superimposed on the mean ROC curve displaying the distribution of ROC curves from 1,000 resamples. **B** Two-graph ROC curve for PVPI at T2 with an intersection point above the 90% sensitivity/specificity line, indicating the absence of an inconclusive grey zone. *AUC* area under the curve, *CI* confidence interval, *PVPI* portal vein pulsatility index, *ROC* receiver operating characteristic, *T1* baseline, *T2* 1 min after passive leg raising, *T4* 2 min post-Ringer’s Lactate

To minimize the impact of outliers and address the small sample size, bootstrapping was employed for ROC curve analysis to evaluate the predictive accuracy of the baseline (T1) and post-PLR (T2) PVPIs in determining congestion status (PVPI ≥ 50% vs. PVPI < 50%) 2 min after Ringer’s Lactate infusion (T4) (Fig. 5, Table 5). The post-PLR PVPI (T2) demonstrated a greater area under the curve (AUC) (0.998) than did the baseline PVPI (T1) (0.902), suggesting better predictive accuracy for congestion. However, despite a mean AUC difference of − 0.095 95%CI (− 0.212 to − 0.005), the result was not statistically significant (*p* = 0.467), likely due to bootstrapped sample variability.

**Table 5:** Predictive performance of bootstrapped PVPI for T4 congestion

PVPI	AUC (95% CI)	Cut off % (95% CI)	SE % (95% CI)	SP % (95% CI)	PPV % (95% CI)	NPV % (95% CI)	*p*-value
T1	0.902 (0.784–0.995)	13.06 (5.5–16.0)	85.3 (66.7–100.0)	94.5 (80.4–100.0)	92.8 (75.0–100.0)	88.71 (64.1–100.0)	< 0.001
T2	0.998 (0.985–1.000)	44.3 (41.0–48.0)	99.0 (91.9–100.0)	98.7 (91.3–100.0)	98.41 (89.5–100.0)	99.2 (93.4–100.0)	< 0.001

*AUC* area under the curve, *CI* confidence interval, *NPV* negative predictive value, *PPV* positive predictive value, *PVPI* portal vein pulsatility index, *SE* sensitivity, *SP* specificity, *T1* baseline, *T2* 1 min after passive leg raising, *T4* 2 min post-Ringer’s Lactate

## Discussion

This exploratory pilot trial investigated 40 adult patients undergoing elective open-heart surgery who required cardiocirculatory optimization within 6 h of ICU admission and were identified as fluid-tolerant preload-responders. The study found that 45% of these patients developed transient systemic venous congestion (PVPI ≥ 50%) after a 7 ml/kg Ringer’s Lactate challenge, suggesting the presence of two distinct phenotypes of right ventricular functional reserve. Post-PLR PVPI demonstrated strong predictive accuracy for this dynamic congestion, indicating that the PLR test could effectively replace the crystalloid challenge. Compared to the non-congested group, the transiently congested group exhibited poorer baseline right ventricular function, lower MPP, and a higher incidence of severe AKI at 7 days. PLR can thus assess both preload and right ventricular functional reserve, with post-PLR PVPI guiding fluid management and potentially suggesting alternative therapies to fluid administration for patients with impaired right ventricles.

Several important considerations from our exploratory analysis should be highlighted in relation to the literature. First, our findings align with those of previous studies showing that the arterial flow effects of a fluid challenge diminish within 5 to 10 min after the infusion is completed [19, 20]. This phenomenon may be due to various mechanisms, including stress-relaxation, reduced sympathetic outflow due to transiently increased cardiac output, fluid redistribution in the splanchnic vasculature, and capillary leakage [21].

Second, while the fluid challenge did not have a lasting impact on arterial flow, it led to a persistently altered portal Doppler profile indicative of venous congestion, specifically in patients who had reduced right ventricular function at baseline. Conversely, PVPI has been identified as an early marker of venous congestion even in healthy volunteers with presumably normal right ventricular function but who failed to exhibit an increase in stroke volume following a 500 ml Ringer’s Lactate challenge [22]. PVPI has been shown to respond not only to preload challenges but also to mixed preload-afterload challenges, such as those induced by incremental PEEP, establishing it as a dynamic marker of the interaction between mechanical ventilation and right heart function [23]. Our study further demonstrated that PVPI is a dynamic marker of right ventricular functional reserve, readily responsive to positional changes.

Third, the mixed-model graph (Fig. 4) illustrating the relationship between LVOT-VTI (a proxy for systemic arterial flow) and PVPI (a proxy for filling pressures) aligns with the Doppler Starling curve described by Kenny et al. [24]. The identical slopes but differing intercepts of the regression lines indicate that, while both groups were fluid-responsive at baseline, their right ventricular reserve differed primarily in its diastolic rather than its systolic component.

Fourth, others have also explored using positional changes to assess diastolic reserve. Choi et al. demonstrated that PLR, by increasing thoracic stressed blood volume, can identify patients with reduced left ventricular diastolic reserve, particularly those with an abnormal relaxation pattern [25].

Fifth, the concept of using positional changes along with venous liver flow profiles to assess cardiovascular reserves has recently been advanced. Bruna et al. showed that preload responsiveness could be predicted by an increase in post-PLR hepatic vein Doppler S-wave velocity [26]. Interestingly, this study reported high specificity but only moderate sensitivity, suggesting that numerous false negatives—patients whose S-wave velocity did not increase after PLR—might actually be preload responders with limited right ventricular diastolic reserve, similar to our patients who exhibited increased PVPI after the PLR test or the 7 ml/kg Ringer’s Lactate challenge.

Lastly, concurrent analysis of LVOT VTI and PVPI profiles (Fig. 3) revealed that, unlike LVOT VTI, PVPI did not return to baseline at T3. This discrepancy is unlikely due to measurement timing, as images were collected and analyzed offline. The most plausible explanation is the physiological difference between systemic arterial (LVOT VTI) and systemic venous (PVPI) events. The dissipation time for each depends on the compartment’s time constant (τ), with systemic venous τ (τ = resistance × compliance) being approximately three times greater than systemic arterial τ, as modeled by Magder et al. [27]. This larger τ in the venous system accounts for the observed lag between arterial and venous responses. Importantly, this lag does not affect our findings. PVPI post-PLR (a *virtual* fluid challenge) at T2 effectively predicted congestion status (PVPI ≥ 50% vs PVPI < 50%) after Ringer’s Lactate infusion (a *real* fluid challenge) at T4. This status delineates two distinct phenotypes of right ventricular diastolic reserve with distinct clinical outcomes. Specifically, the T4 PVPI increase likely captures the added effect of the crystalloid challenge and the delayed post-PLR response. However, this does not alter the conclusion that one group exhibited a more severe sum of effects compared to the other, which was associated with a higher incidence of severe AKI.

Our study has several limitations that should be acknowledged. First, fluid intolerance due to systemic venous congestion and fluid responsiveness were defined dichotomously using a single cutoff, a PVPI ≥ 50 and a post-PLR LVOT-VTI change ≥ 12%, respectively. However, congestion is a biological process that likely exists on a continuum of severity, with increasing risks for AKI and other complications [28]. Similarly, the binary definition of fluid responsiveness is limited by confounding factors such as impaired right ventricular function and elevated intra-abdominal pressure, which are better accounted for when a continuous definition, using the mean systemic filling pressure analogue, is applied [29].

Second, although previous studies have shown that a combined assessment—including IVC, hepatic, portal, and intrarenal veins—enhances the specificity of venous congestion evaluation, we aimed to simplify this process by focusing solely on PVPI [30]. PVPIs are easier to acquire, less time-consuming, and more practical for integration into bedside clinical care. Furthermore, PVPI correlates well with intrarenal Doppler indices [7] and, unlike intrarenal and hepatic indices, it can be interpreted both categorically (e.g., PVPI ≥ 50%) and as a continuous variable, providing a more nuanced assessment of congestion severity.

Third, while echocardiographic data were not intended to influence patient management, the lack of blinding for the ICU intensivist may have subtly influenced postoperative outcomes.

Finally, as an exploratory pilot trial designed to generate hypotheses, a formal sample size calculation was not conducted, which limits the generalizability of our findings. The results should be interpreted with caution, and further research with larger, adequately powered studies is needed to validate these preliminary observations and to explore their implications in a broader clinical context.

## Conclusion

This study shows that post-PLR PVPI effectively predicts fluid-induced systemic venous congestion in fluid-tolerant preload-responders, identifying patients with poor right ventricular diastolic reserve. This supports its use in guiding fluid management to prevent unnecessary administration and related complications in cardiac surgery patients.

## Data Availability

The data supporting the findings of this study are available from the corresponding author, Cosmin Balan, upon reasonable request.

## Abbreviations

ACC: Aortic cross-clamp
AKI: Acute kidney injury
AUC: Area under the curve
BMI: Body mass index
CABG: Coronary artery bypass graft
CI: Confidence interval
CPB: Cardiopulmonary bypass
CrCl: Creatinine clearance
CVP: Central venous pressure
ΔCO_2_: Difference between central venous partial pressure of carbon dioxide (PCO_2_) and arterial PCO_2_
Dmaximum: Maximum diameter of inferior vena cava
EuroSCORE II: European System for Cardiac Operative Risk Evaluation II
ICU LOS: Intensive care unit length of stay
IVC (DI): Inferior vena cava (distensibility index)
KDIGO: Kidney Disease: Improving Global Outcomes
LSA: Least squares adjusted
LVEF: Left ventricular ejection fraction
LVEDA: Left ventricular end-diastolic area
LVOT-VTI: Left ventricular outflow tract velocity time integral
MAP: Mean arterial pressure
MMRM: Mixed model for repeated measures
MPP: Mean perfusion pressure
NPV: Negative predictive value
PPV: Positive predictive value
PVPI: Portal vein pulsatility index
ρ (RHO): Repeated measure correlation coefficient
RMCORR: Repeated measure correlation
ROC: Receiver operating characteristic
RVEDA: Right ventricular end-diastolic area
RVFAC: Right ventricular fractional area change
RV S’: Tissue Doppler-derived tricuspid lateral annular systolic velocity
SAPS II: Simplified Acute Physiology Score II
ScVO_2_: Central venous oxygen saturation
SE: Sensitivity
SP: Specificity
TAPSE: Tricuspid annular plane systolic excursion
T1: Baseline
T2: 1 min after passive leg raising
T3: 2 min after return to the semi-recumbent position
T4: 2 min post-Ringer’s Lactate
T5: 20 min post-Ringer’s Lactate
